# Percutaneous endoscopic gastrostomy site metastasis from head and neck squamous cell carcinoma: case series and literature review

**DOI:** 10.1186/1916-0216-42-20

**Published:** 2013-02-28

**Authors:** Andrew T Huang, Alexandros Georgolios, Sasa Espino, Brian Kaplan, James Neifeld, Evan R Reiter

**Affiliations:** 1 Department of Otolaryngology – Head and Neck Surgery, Virginia Commonwealth University Massey Cancer Center, Richmond, VA, USA; 2 Commonwealth University School of Medicine, Richmond, VA, USA; 3 Department of Surgery, Virginia Commonwealth University Massey Cancer Center, Richmond, VA, USA

**Keywords:** Percutaneous endoscopic gastrostomy, Head and neck cancer, PEG, Metastasis, Prophylactic irradiation

## Abstract

**Objectives:**

To present our experience with head and neck squamous cell carcinoma (HNSCC) seeding of percutaneous endoscopic gastrostomy (PEG) sites and to review all reported cases to identify risk factors and develop strategies for complication avoidance.

**Materials and methods:**

The records of 4 patients with PEG site metastasis from HNSCC were identified from the authors’ institution. Thirty-eight further cases were reviewed following a PubMed search and evaluation of references in pertinent articles.

**Results:**

Review of 42 cases revealed the average time from PEG to diagnosis of metastatic disease to be 8 months. Average time to death from detection of PEG disease was 5.9 months. One-year survival following PEG metastasis was 35.5% with an overall mortality of 87.1%.

**Conclusion:**

PEG site metastatic disease portends a poor prognosis. Early detection and aggressive therapy may provide a chance of cure. Changes in PEG technique or in timing of adjunctive therapies are possible avenues in further research to prevent this complication.

## Introduction

The technique of percutaneous endoscopic gastrostomy (PEG) was originally introduced by Gauderer and Ponsky in 1980 at a meeting of the American Society of Gastrointestinal Endoscopy as a novel method for obtaining long-term enteral access in neurologically-debilitated patients [1]. Since its inception, extensive validation of the safety and efficacy of PEG has been reported by general surgeons, gastroenterologists and otolaryngologists. Today the PEG technique has largely superseded the use of nasogastric tubes and open gastrostomy tube placement for prolonged nutritional support due to reported reductions in major complications, patient discomfort, days spent in the hospital, and cost [2-5].

Patients with head and neck squamous cell carcinoma (HNSCC) represent a distinct group of patients requiring alternate means for nutrition. It is estimated that 200,000 PEGs are performed in the United States every year, with head and neck cancer patients comprising 5% of procedures [2]. In fact, 33-69% of patients undergoing definitive chemoradiotherapy for upper aerodigestive tract malignancies ultimately require PEG tube placement [6,7]. As the cumulative number of PEG’s has increased, new complications, previously unforeseen, have been described. One such complication specific to head and neck cancer, PEG-site implantation of metastatic disease, has gained significant notoriety in the recent literature. However, as this occurrence is rare, the literature has largely been limited to isolated case reports. The small number of reported cases and lack of existing large patient series or prospective studies has precluded adequate examination of this phenomenon. In an effort to better identify the pertinent risk factors for this potentially fatal complication, we present our institutional experience of four cases of metastatic spread of HNSCC to PEG-sites, the largest series in the literature to date, and also systematically review all cases of PEG site metastases from HNSCC previously reported in the literature.

## Methods

Four patients diagnosed with PEG-site metastases occurring after treatment for HNSCC at the Virginia Commonwealth University Health System were retrospectively identified. Informed consent was obtained from all patients for publication of this report and any accompanying images. Charts were reviewed for pertinent history including patient demographics, tumor location and staging, timing of PEG tube placement in relation to primary oncologic therapy, method of PEG tube insertion, length of time until diagnosis of PEG tube metastasis, and modality and outcome of PEG site metastasis treatment.

To assess the current literature on PEG site metastases arising in patients with HNSCC, a MEDLINE search was performed through the United States National Library of Medicine’s “PubMed” online database. A total of 111 papers were obtained using the search terms “Gastrostomy” and “Metastasis” with results limited to the English language. Esophageal primary cancers were excluded from review. Case reports, series and reviews were identified and their citations examined for further resources. Thirty-four publications [3,4,8-39] were identified, comprising a total of thirty-eight patients. These publications were reviewed to extract the historical information outlined above.

## Results

### Case presentation 1

A 69 year-old male with forty-eight pack-year history of smoking presented to our institution with a T2N2aM0 SCCA of the right piriform sinus. One month prior to initiation of primary chemoradiotherapy a PEG tube was placed by the gastroenterology service using the Gauderer-Ponsky technique. Treatment included radiation to a maximum tumor dose of 70 Gy, administered with adjuvant carboplatin and taxotere. Post treatment endoscopy and whole body PET scan suggested persistent disease only in a residual right neck mass. He underwent salvage right selective neck dissection five months following cessation of chemoradiation, with final pathology revealing only fibrosis with no viable malignant cells. Approximately five months after completing treatment, as he was tolerating a regular oral diet without dysphagia, his PEG tube was removed and the site promptly healed. Fourteen months after neck dissection, a total of twenty months following chemoradiation and twenty-two months following PEG placement, repeat whole body PET scan revealed metastatic foci to the adrenal glands, liver, and left anterior abdominal wall. CT-guided biopsies of the abdominal wall mass revealed poorly-differentiated squamous cell carcinoma for which he underwent palliative chemotherapy. He subsequently developed diffuse, painful bony metastases which were treated with palliative radiotherapy. Eight months after diagnosis of the PEG metastasis, he died following a stroke.

### Case presentation 2

A 77 year-old male with a history of alcohol abuse and over twenty pack-year history of smoking was treated for a T3N1M0 SCCA of the right supraglottic larynx. One week prior to initiation of primary chemoradiotherapy, he underwent a routine Gauderer-Ponskey PEG by the gastroenterology service. The patient was treated with IMRT to a total dose of 70 Gy with concurrent weekly cisplatin. Post-treatment endoscopy, four months following treatment conclusion, revealed no evidence of persistent disease in the upper aerodigestive tract. The patient remained PEG-tube dependent for the majority of his nutritional needs due to dysphagia caused by extensive hypopharyngeal scarring. Fourteen months after completing treatment he was admitted to the hospital for constipation and acute renal failure. An upper GI series showed a large gastric ulcer at the site of his PEG which was pathologically confirmed as squamous cell carcinoma on esophagogastroduodenoscopy performed seventeen months after PEG placement. Computed tomography of the chest and whole body PET scan showed no other foci of metastatic disease. The patient subsequently underwent exploratory laparotomy for resection of his disease. However, intraoperatively the mass was noted to be 8 cm in maximal diameter with invasion of the colon, mesentery and pancreas, thus precluding adequate resection. His PEG tube was removed and replaced through a separate gastrostomy in normal stomach prior to wound closure. Seven weeks after attempted resection, five months following diagnosis of PEG site metastasis and twenty months after completing primary chemoradiotherapy, he died while in hospice care.

### Case presentation 3

**Figure 1:**
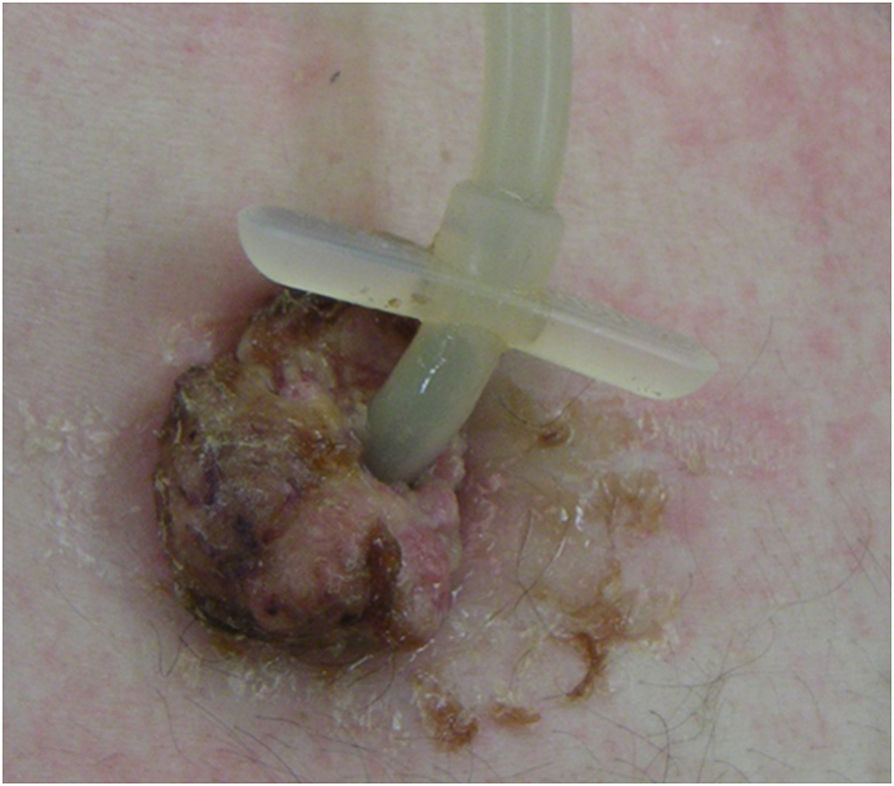
Pre-operative view of abdominal wall component of PEG-site metastasis in Patient 3.

**Figure 2:**
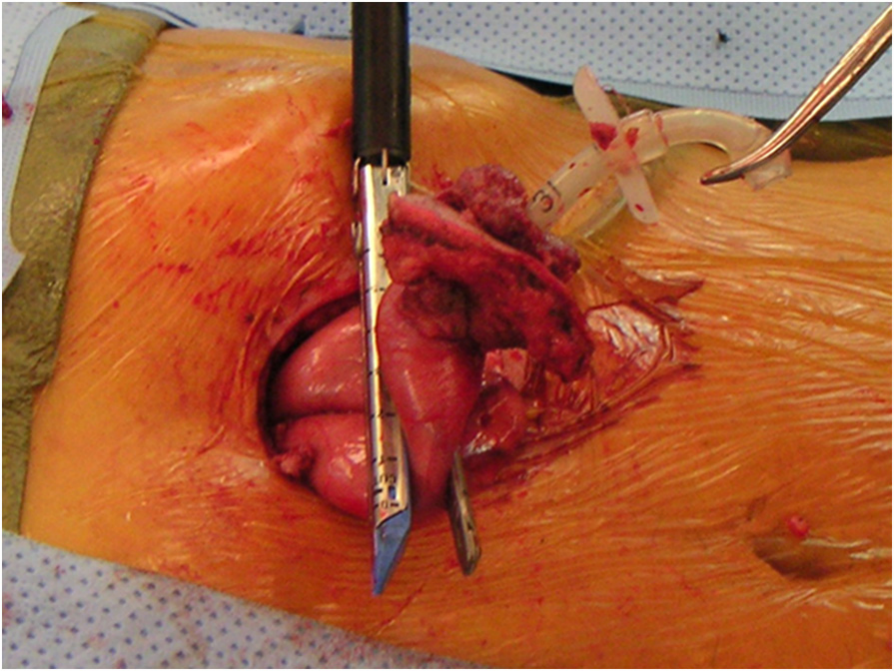
Intra-operative view of PEG tract metastasis in patient 3, with gross tumor extending from the abdominal wall to the stomach (in jaws of endo stapler).

A 46 year-old male with a history of tobacco abuse was diagnosed with T4N1M0 SCCA of the right retromolar trigone. The patient elected for primary chemoradiation therapy and had a PEG placed by the gastroenterology service using the Gauderer-Ponsky technique one month prior to initiation of treatment. He received IMRT to a total dose of 70 Gy with concurrent cisplatin chemotherapy. The patient’s recovery was complicated by osteoradionecrosis of the mandible which was treated successfully with hyperbaric oxygen therapy. Seven months following treatment and nine months following PEG placement, the patient presented with an abdominal wall mass surrounding his PEG tract (Figure 1). Biopsies revealed squamous cell carcinoma, and whole body PET scan demonstrated no other foci of metastatic disease. The patient underwent wide local excision of the lesion, including full-thickness abdominal wall resection and partial gastrectomy (Figure 2). Final pathologic examination revealed clear margins from the resected specimen. Eight months after his abdominal procedure, chest CT revealed a right upper lobe mass, which was confirmed as a new focus of metastatic squamous cell carcinoma. A right upper lobe wedge resection was completed by the thoracic surgical service followed by 36 Gy of adjuvant radiotherapy. Three months after completion of lung radiotherapy, repeat CT scan showed pleural metastases and recurrence at the primary site involving the mandible and tongue base. The patient underwent three cycles of palliative chemotherapy with taxotere, but died of his cancer 2 months after conclusion of therapy, nineteen months following discovery of the PEG metastasis.

### Case presentation 4

A 64 year-old male with a prior history of tobacco abuse was diagnosed with T1N2cM0 SCCA of the right base of tongue. In preparation for treatment, a PEG tube was placed by the Gauderer-Ponsky technique just prior to initiation of therapy. He received primary chemoradiation to a total tumor dose of 70 Gy with concurrent cisplatin chemotherapy. Seven months after finishing treatment, he was found to have recurrent disease at the tongue base and neck in addition to an abdominal wall metastasis surrounding his PEG tract. The patient refused surgical management of the primary site recurrence, and instead underwent a modified radical neck dissection for the right neck disease with an additional 60 Gy radiotherapy directed at the tongue base. At the time of neck dissection, the abdominal wall metastasis was excised with a wide margin of the involved stomach, and a revision open gastrostomy performed. Final pathologic review revealed clear margins. Post-treatment endoscopy and biopsy of the patient’s tongue has not demonstrated recurrent cancer a total of twenty-one months after initial treatment, eleven months from primary site re-irradiation, and 14 months following PEG site recurrence excision.

## Literature review

A total of 38 distinct cases of PEG site metastases associated with HNSCC were identified in the English literature since 1989 from the PubMed Database. These reports, combined with the current series, bring the total number of reported PEG site metastases from HNSCC to 42. Patient demographics, tumor staging, data regarding timing of PEG placement, occurrence of metastatic lesion(s), and treatment outcome are reported in Table 1. The average age on presentation was 57.6 years; 82.9% of patients were male. The oropharynx (40%) was the most common primary tumor site, followed by hypopharynx (29%), oral cavity (17%), and larynx (14%). Staging information was available in 37 of the 42 patients, with 35 (94.6%) initially presenting with advanced (stage III or IV) disease. No patients in the cases reviewed had known distant metastatic disease on initial presentation.

**Table 1:** Reported cases of squamous cell carcinoma implantation at a PEG tube site

**Author**	**Age**	**Sex**	**Site of primary disease**	**TNM stage**	**Treatment of primary disease**	**Timing of PEG placement (before or after primary therapy)**	**Time from PEG placement to stomal metastasis (months)**	**Other concurrent treatment failure sites**	**Treatment of PEG-site metastasis**	**Length of survival after PEG metastasis diagnosis (months)**
Adelson [17]	63	M	Oropharynx	NA	Surgery	Before	6	Lung, liver	Chemo	4
Ananth [3]	55	M	Oral Cavity	T1N2aM0	Surgery + XRT	Before	3.5	NCR	Surgery + Chemo	NED
Balakrishnan [18]	50	M	Larynx	NA	Surgery	Before	NA	NCR	Surgery	NA
Becker [19]	49	M	Hypopharynx	T4N3M0	XRT	Before	3	Lung	Surgery	5
Bhama [20]	51	M	Hypopharynx	T2N1M0	XRT	Before	3.5	Liver, pelvis	Surgery	4
Bushnell [9]	68	M	Supraglottis	T4N2bM0	Surgery + XRT	After	14	Lung	None	18
Chatni [10]	53	F	Hypopharynx	NA	XRT	After	5	Neck	None	NA
Coletti [21]	65	M	Oral Cavity	T4N2cM0	ChemoXRT	Before	9	NCR	ChemoXRT	2
Cossentino [22]	62	M	Oropharynx	T4N2M0	Surgery + XRT	Before	8	Lung	None	2
	66	M	Oropharynx	T3N1M0	XRT	Before	9	NA	None	NA
Cruz [23]	58	M	Oral Cavity	T4N0M0	XRT	Before	5	NA	Surgery + Chemo	4
	48	M	Hypopharynx	T4N3M0	Surgery + ChemoXRT	Before	6	NA	Surgery	4
Daniels [24]	56	M	Oral Cavity	TxN+M0	Surgery + XRT	Before	3	NA	Chemo + Surgery	NA
Douglas [15]	45	M	Oropharynx	T4N3M0	XRT	Before	3.5	NCR	XRT	9
Hawkin [8]	68	M	Oropharynx	T3N0M0	XRT	Before	14	NCR	None	1
Huang [25]	53	M	Oropharynx	T4N2aM0	Surgery + XRT	Before	6	NA	NA	NA
Kurdow [26]	75	F	Hypopharynx	T4N0M0	XRT	Before	4	NA	NA	NA
Laccourreye [11]	65	M	Hypopharynx	T3N0M0	ChemoXRT	After	11	Liver	XRT	4
Lee [27]	41	M	Oropharynx	T4N2bM0	Surgery + ChemoXRT	Before	13	Liver, spleen	None	1
Lim [28]	51	M	Oropharynx	T4N1M0	Surgery + XRT	Before	8	Lung	Surgery	NA
Lin [13]	56	M	Oropharynx	T4N0M0	Surgery + XRT	Before	5	NCR	NA	2
Maccabee [29]	63	F	Hypopharynx	T4N1M0	ChemoXRT	Before	5	Primary site	None	3
Meurer [30]	45	M	Oropharynx	T3N1M0	Surgery	Before	12	Lung	ChemoXRT	17
	76	F	Oropharynx	T2N0M0	XRT	Before	13	Lung	Surgery	16
Mincheff [4]	59	M	Oropharynx	T4N2bM0	Surgery + XRT	NA	4	NCR	Surgery + XRT	NA (Hospice)
Potochny [31]	44	M	Hypopharynx	T2N2M0	Surgery + XRT	Before	9	NCR	Surgery	NED
Preyer [14]	72	M	Oropharynx	T4N2cM0	ChemoXRT	Before	3	Lung	None	3
Purandare [32]	50	F	Oropharynx	T3N2M0	Surgery	NA	9	Primary site, neck	NA	NA
	62	M	Oral Cavity	T2N0M0	Surgery	NA	15	Primary site	NA	NA
Schiano [12]	43	M	Hypopharynx	T4NxM0	Chemo	After	4	Primary site, neck	NA	NA
Schneider [33]	61	F	Oropharynx	T4N0M0	Surgery + XRT	Before	10	NCR	Surgery	NED
Sharma [34]	40	M	Oral Cavity	T4N3M0	ChemoXRT	Before	6	Primary site	Surgery + Chemo	NA (Hospice)
Siddiqi [35]	56	F	Supraglottis	T3N2bM0	ChemoXRT	Before	7	NA	NA	2
Sinclair [36]	61	M	Oropharynx	T2N1M0	XRT	Before	5	Left axilla	Surgery + ChemoXRT	NA (recurrent disease detected)
Thakore [37]	50	M	Larynx	NA	Surgery + ChemoXRT	NA	NA	Lung, hip, brain	ChemoXRT	6
Thorburn [38]	56	M	Supraglottis	T4N3M0	XRT	Before	11	NA	NA	1
Tucker [16]	NA	NA	Hypopharynx	NA	ChemoXRT	Before	3	NA	NA	NA
Van erpecum [39]	69	M	Hypopharynx	T4N0M0	XRT	Before	10	NCR	XRT + Surgery	1
Current series	69	M	Hypopharynx	T2N2aM0	ChemoXRT	Before	22	Adrenals, liver, lung	Chemo XRT	8
	77	M	Supraglottis	T3N1M0	ChemoXRT	Before	16	NCR	Chemo	5
	46	M	Oral Cavity	T4N1M0	ChemoXRT	Before	8	NCR	Surgery	19
	64	M	Oropharynx	T1N2cM0	XRT	Before	7	Primary site, neck	Surgery	NED
Total cases	42									

Abbreviations: *NA*, not available; *Chemo*, Chemotherapy; *XRT*, radiation therapy; *NCR*, no concurrent recurrence; *NED*, no evidence of disease.

The method of PEG tube insertion was documented in 29 cases, with 28 (96.6%) reporting use of the Gauderer-Ponsky (“pull”) technique and one [8] using the radiologic-assisted method. Thirty-eight cases documented the timing of PEG tube placement in relation to primary oncologic therapy. Thirty-four (89.5%) of these patients had PEG tubes placed prior to definitive cancer therapy, while 4 (10.5%) had tubes placed following primary treatment failure in preparation for further treatment. In all of the latter 4 cases, there was persistent or recurrent disease at the primary site [9-12]. The time from PEG placement to diagnosis of PEG site metastatic disease was described for 40 patients, with the mean duration until diagnosis of PEG site disease being 7.96 months (3 – 22 months). Patient outcome after treatment of metastatic lesions was found in thirty-one cases. Twenty-four patients had documented death from disease with average time from identification of PEG metastasis to death being 5.9 months (1 – 19 months). Three patients were reported as hospice care subjects with active disease present, and are presumed to have succumbed to their disease for purposes of our analysis. Four patients, as of the publication of the associated reports, were free of disease, however follow up time was not documented except in the current series with one patient surviving free of disease after 14 months. Thus, overall mortality was calculated to be a minimum of 87.1% with one-year survival of only 35.5%, and no documented survivors beyond 19 months from diagnosis of PEG site metastasis reported. The presence of recurrent locoregional or distant metastatic disease concurrent with PEG site metastasis was reported in 21 (63.6%) of the thirty-three patients for which disease status was clearly delineated with the most common sites of concurrent metastatic foci being the lung and liver.

## Discussion

Chronic malnutrition affects approximately 20-57% of patients with head and neck cancer [40]. Increased catabolism, anorexia, dysphagia, odynophagia, and aspiration are several factors leading to cancer cachexia and malnutrition in this population [41]. Weight loss prior to and during head and neck cancer treatment portends a variety of treatment difficulties. Van Bokhorst-De Van Der Schueren et al [42] found that a greater than 10% weight loss over a six month period was the most powerful predictor of major post-operative complications. With approximately 54% of head and neck cancer patients admitting to restricted diets of soft or pureed foods [43], other means of nutritional support are frequently necessary to prevent poor treatment outcomes. Due to this, the benefits of enteral nutritional support as an adjunct in the management of head and neck cancer have become well established.

Enteral feeding by open gastrostomy was first introduced in 1875 and was often considered a measure of last resort due to the up to 50% risk of major complications associated with the procedure [44,45]. Although complication rates for open gastrostomy have vastly improved in the modern era, the introduction of the percutaneous endoscopic gastrostomy technique provided a less invasive and easily reproducible method for providing prolonged enteral support for medically complicated patients. Various methods of PEG tube placement have since been devised, with the most commonly referenced being the Gauderer-Ponsky, Sacks-Vine [46], Russell [47], and radiologic-assisted techniques [48]. Common to all methods of PEG, however, is the insertion of either an endoscope, nasogastric tube, or the feeding tube itself through the oral cavity and pharynx to the stomach for gastric visualization and/or insufflation. Reported benefits of PEG over open gastrostomy placement include decreased pain, abdominal complications, and cost [4,13,44]. Nasogastric feeding, although easily placed and relatively non-invasive, has its own risks, including gastroesophageal reflux, nasal alar erosion and deformity, laryngeal irritation, inadvertent tube removal, and sinusitis, and is thus generally not considered a viable long-term option for enteral feeding [44,45].

After Preyer [14] published the first case of PEG site metastasis from an oropharyngeal primary HNSCC in 1989, the complication of incidental seeding of the gastric or abdominal wall following PEG has become a developing concern. Several theories of the pathogenesis of this occurrence have been proposed, including direct implantation of malignancy at the time of tube placement, physiologic shedding of malignant cells into the alimentary tract with seeding of the PEG site after tube placement, and hematogenous spread with selective preference of circulating tumor cells to implant at the traumatized tissue of the PEG wound [15,49].

Although the incidence of PEG site metastases is low, estimated at 0.5 – 3% [2,4,13,50], survival outcomes indicate that this complication carries a grave prognosis. The estimated survival rate of 12.9% shown in the present review, although seemingly better than that reported for other sites of distant metastatic disease in HNSCC (1–6.5%) [51,52], is likely a gross overestimation as available follow up in the case reports reviewed was well below five years. In addition, 64% of patients diagnosed with PEG site disease either had simultaneous or subsequent locoregional or distant metastatic disease, suggesting that PEG site metastases may be a marker of aggressive tumor behavior.

Presentations of PEG site metastasis include incidental imaging findings on metastatic work-up, vague abdominal discomfort, constipation, grossly evident tumor emanating from the abdominal wall, ulceration, and persistent stomal drainage [2]. Some of these findings lack specificity, however, due to the fact that common complications of PEG, including stomal leakage of gastric secretions and formation of granulation tissue, may mimic tumor [45]. Thus, knowledge of this complication and continued vigilance by all members of the head and neck oncologic team are critical to early detection, which might provide some hope for curative treatment.

Based upon the theories for the pathogenesis of PEG site metastases, especially that of direct tumor implantation, many procedural recommendations have been made for its avoidance. As the majority of reported cases of PEG site metastasis are associated with the Gauderer-Ponsky technique (96.6%), authors have suggested use of alternate techniques, such as Russell (transabdominal introduction of gastrostomy tube under endoscopic visualization), Sacks-Vine (blind pulling of the feeding tube through the abdominal wall via the mouth under nasogastric stomach insufflation), or radiologic-assisted to avoid passage of the feeding tube, endoscope, or both, past the site of the tumor [53]. The Russell, or “push”, technique has been suggested to be a preferable compromise between ease of performance and risk of procedure for PEG placement in HNSCC patients [2,16,52]. Although this technique obviates the need to pull the feeding tube through the oral cavity and pharynx, an endoscope is still required for visualization within the stomach during feeding tube insertion through the abdominal wall. In 2003, Tucker et al [16] reviewed 79 HNSCC patients undergoing PEG, 29 via the push technique and 50 via the pull technique. The authors found a 0% complication rate with push PEGs compared to 30% in those undergoing pull PEGs. One patient undergoing pull technique PEG presented with a PEG site metastasis, but the small study population size and overall low rate of PEG metastasis makes this result difficult to interpret when comparing the two methods. Theoretically, however, the smaller caliber and maneuverability of the endoscope should allow less trauma to the tumor surface than a blindly passed feeding tube, as is required with pull techniques such as Gauderer Ponsky or Sacks-Vine. The same argument has been made regarding the percutaneous placement of feeding tubes under fluoroscopic guidance in cases of HNSCC requiring enteral feeding [50,53]. The paucity of published reports of PEG site metastasis from HNSCC using the percutaneous radiologic-assisted gastrostomy or the Russell technique tends to suggest that direct implantation of malignant cells at time of tube placement is the most plausible explanation for PEG site seeding. Data from the current series may also be interpreted to indirectly support this theory. The shorter time interval from PEG placement to diagnosis of PEG site disease (7.96 months) compared with timing of presentation of distant metastases established via hematogenous seeding (median 12 months) [51] can be argued to reflect a larger initial metastatic deposit, as would be expected from implantation of tumor liberated by direct trauma to an existing tumor mass. Douglas et al [15], using tumor kinetic assumptions, hypothesized a bimodal distribution of PEG metastases with those appearing quickly most likely representing direct tumor implantation, and those appearing after a prolonged period (> 12 months) being a result of hematogenous spread. Of the 39 cases in which time from PEG placement to identification of PEG metastasis was reported, only 6 had intervals greater than 12 months. The fact that the only reported case of PEG site metastasis following fluoroscopic-guidance [8] presented 14 months after tube placement, also weakly supports hematogenous metastatic implantation. Lastly, use of open gastrostomy, which also avoids the need for passage of an endoscope or feeding tube past the tumor site, may be a reasonable option in select patients, such as those with bulky tumors undergoing general anesthesia for other indications.

In addition to surgical alterations to prevent metastatic complications, changes in procedure timing and adjunctive modalities should also be considered. Alteration in timing of PEG tube placement in relation to HNSCC therapy has been analyzed as a potential strategy for prevention of stomal metastasis. The concept of direct tumor seeding has been implicated in other phenomena, namely stomal recurrence after total laryngectomy [54,55]. Analysis of seventeen cases of peristomal recurrence of squamous cell carcinoma following total laryngectomy found pre-laryngectomy tracheostomy to be the sole significant risk factor for occurrence, with direct stomal implantation of tumor cells the hypothesized mode of transmission [55]. Although early advocates of PEG in head and neck cancer patients recommended pre-treatment tube placement to provide earlier nutritional support [56], our review revealed that 89% of PEG site metastases occurred in patients undergoing PEG prior to initiation of definitive therapy. With this in mind, future research may be indicated to assess the benefit of deferring PEG placement until after initiation of radiotherapy or tumor resection. Similarly, prophylactic irradiation of the PEG site, especially in patients with bulky pharyngeal disease is an option shown to be of merit in other malignancies and tumor implantation locations. Prophylactic radiation given before pre-laryngectomy tracheostomy has been shown to decrease the incidence of peristomal recurrence, although at the cost of increased regional failure [54]. For small-cell lung cancer, prophylactic cranial irradiation is well-established as standard of care to prevent metastasis and improve 3-year survival [57]. Prospective studies to investigate the use of prophylactic PEG irradiation will be needed to assess the feasibility and benefit in the HNSCC patient population. Lastly, tumor-cell attachment inhibitors such as dispase have been shown to block metastatic implantation at surgical wound sites and may hold promise for the prevention of implantation at PEG sites [58].

## Conclusion

Enteral feeding is an important adjunct in the treatment of head and neck cancer that has been shown to improve outcomes and patient quality of life. Although a less invasive procedure than open gastrostomy tube placement, PEG has its own complications including the development of metastatic tumor deposits at the gastrostomy site. As this occurrence seems to be limited to use of standard “pull” techniques of PEG placement, such as that of Gauderer-Ponsky, and is more common with advanced stage tumors and pretreatment placement, alternate timing and means of tube placement should be considered, especially with bulky tumors. Familiarity with this complication and careful monitoring by the head and neck oncologic team may allow early detection and treatment. Further research is warranted to evaluate the preventive impact of alternate gastrostomy placement techniques, the timing of PEG placement with respect to initiation of radiotherapy, and the use of chemotherapeutic agents.

## Competing interests

The authors declare they have no competing interests.

## Authors’ contributions

ATH composed the manuscript and analyzed the data. AG and SE analyzed data and participated in manuscript preparation. BK, JN, and ERR participated in manuscript preparation. All authors read and approved the final manuscript.
